# The Selective 3-MST Inhibitor I3MT-3 Works as a Potent Caspase-1 Inhibitor

**DOI:** 10.3390/ijms26052237

**Published:** 2025-03-02

**Authors:** Kohei Otani, Ryuto Komatsu, Takuya Noguchi, Wakana Suzuki, Yusuke Hirata, Atsushi Matsuzawa

**Affiliations:** Laboratory of Health Chemistry, Graduate School of Pharmaceutical Sciences, Tohoku University, Sendai 980-8578, Japan

**Keywords:** inflammasomes, caspase-1, anti-inflammatory drugs

## Abstract

I3MT-3 (HMPSNE) has been identified as a selective inhibitor of the supersulfide-producing enzyme 3-MST. In this study, we found that I3MT-3 inhibits inflammatory responses, including the secretion of the pro-inflammatory cytokine interleukin-1β (IL-1β) and inflammatory cell death pyroptosis, induced by the activation of the inflammasomes composed of NLRP1, NLRP3, or AIM2. However, interestingly, the knockdown of 3-MST did not affect the activation of the inflammasomes, suggesting that the inhibitory effect of I3MT-3 on inflammasome activation is mediated by alternative ways rather than the inhibition of 3-MST. Interestingly, an in vitro caspase assay revealed that I3MT-3 directly inhibits caspase-1 activation, and molecular docking simulations raised the possibility that the pyrimidone ring in I3MT-3 stabilizes direct interaction of I3MT-3 with caspase-1. Taken together, our data suggest that I3MT-3 inhibits inflammasome activation by targeting caspase-1, and show I3MT-3 as a potent inhibitor of caspase-1.

## 1. Introduction

Caspase-1 is one of the cysteine proteases that play an important role in inflammatory responses [1]. In particular, caspase-1 cleaves and thereby activates pro-inflammatory cytokines, such as IL-1β and IL-18, which contribute to the elimination of pathogens and the rapid repair of damaged tissues, and enhance the host immune response. On the other hand, the overactivation of caspase-1 causes an excessive release of IL-1β, leading to both acute and chronic inflammation which can develop various inflammatory diseases. In general, caspase-1 activation is governed by multiprotein signaling complexes called inflammasomes [1,2]. The inflammasomes are mainly composed of the sensor proteins, the adaptor protein apoptosis-associated speck-like protein containing a CARD (ASC), and caspase-1. The nod-like receptor proteins (NLRP) 1 and 3, and absent in melanoma 2 (AIM2) work as the sensor proteins of the inflammasomes that are activated by both microbial-associated molecular patterns (PAMPs), and they host damage-associated molecular patterns (DAMPs) as well as a wide variety of chemicals [3,4,5,6]. The activated sensor proteins promote the formation of the inflammasomes that trigger the activation of caspase-1, and consequently caspase-1-dependent inflammatory responses, such as mature IL-1β release and inflammatory cell death pyroptosis. On the other hand, the aberrant activation of the inflammasomes contributes to the development of chronic inflammation and autoimmune diseases, and thus proper control of inflammasome activity is of great clinical importance [7,8,9]. In particular, the development of drugs that suppress inflammatory responses induced by multiple inflammasomes has attracted attention as a new therapeutic strategy for inflammasome-related diseases.

In recent years, it has been suggested that supersulfide is involved in many inflammatory response signals, and its role in inflammatory responses has attracted attention [10]. Accumulating evidence demonstrates that several enzymes, including cystathionine γ-lyase (CSE), 3-mercaptopyruvate sulfurtransferase (3-MST), cysteinyl-tRNA synthetase, and cystathionine β-synthase (CBS), are involved in endogenous supersulfide production [11,12,13,14,15]. Based on these findings, inhibitors targeting supersulfide-producing enzymes have been developed. For example, propargylglycine (PAG) is known as an inhibitor of CSE, and aminooxyacetic acid (AOAA) and trifluoroalanine (TFA) are known as inhibitors of CBS [16,17,18]. Meanwhile, an isothiazole derivative, I3MT-3, has been recently reported as a specific inhibitor of 3-MST [19]. These inhibitors selectively suppress the activity of each enzyme, making them useful tools for analyzing the physiological and pathological effects associated with supersulfide production. In this study, we used I3MT-3 to evaluate its effect on inflammatory responses, and, interestingly, we found that I3MT-3 acts as an inflammasome inhibitor by inhibiting caspase-1 activation.

## 2. Results

### 2.1. I3MT-3 Inhibits Inflammatory Responses Induced by the Inflammasomes

**Figure 1:**
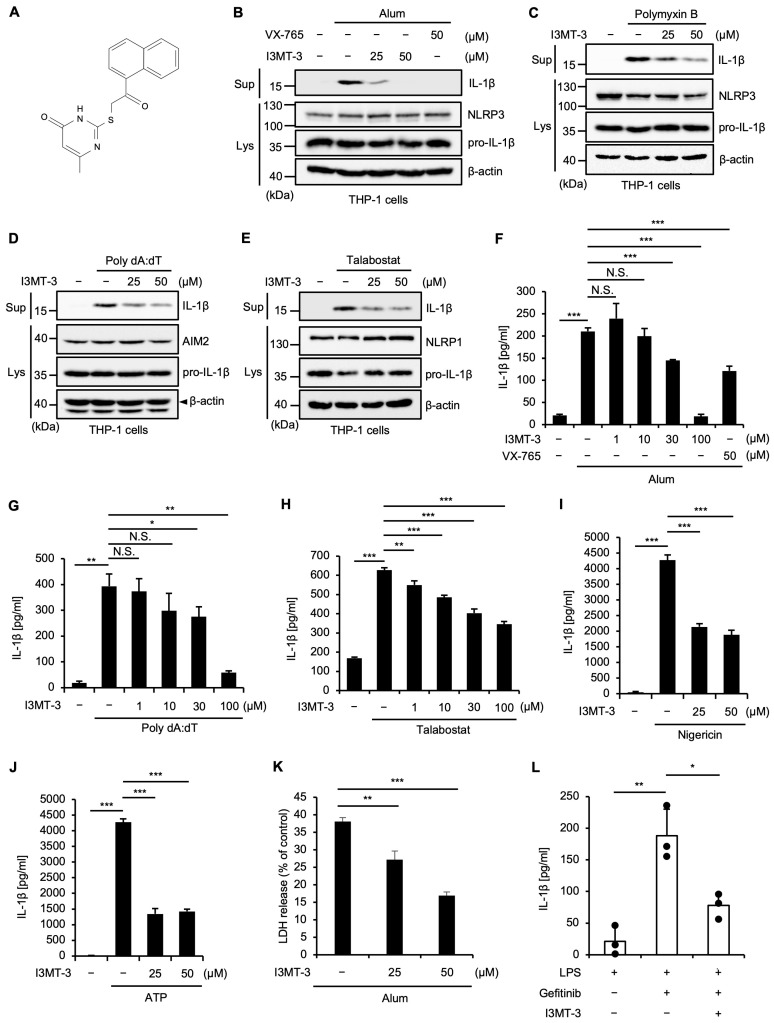
I3MT-3 inhibits inflammatory responses induced by the inflammasomes. (**A**) Structural formula of I3MT-3. (**B**) PMA-differentiated THP-1 cells were cotreated with the indicated concentrations of I3MT-3 or VX-765 (a caspase-1 inhibitor used as a positive control that inhibits Alum-induced IL-1β release), and 200 μg/mL Alum for 4 h [22]. Cell-free supernatants (Sup) and cell lysates were subjected to immunoblotting with the indicated antibodies. (**C**) PMA-differentiated THP-1 cells were pretreated with the indicated concentrations of I3MT-3 for 1 h and then treated with 40 μg/mL PMB for 2 h. Cell-free supernatants (Sup) and cell lysates were subjected to immunoblotting with the indicated antibodies. (**D**) PMA-differentiated THP-1 cells were cotreated with the indicated concentrations of I3MT-3 and 3 μg/mL Poly dA:dT for 4 h. Cell-free supernatants (Sup) and cell lysates were subjected to immunoblotting with the indicated antibodies. (**E**) PMA-differentiated THP-1 cells were pretreated with the indicated concentrations of I3MT-3 for 1 h and then treated with 1 μM Talabostat for 3 h. Cell-free supernatants (Sup) and cell lysates were subjected to immunoblotting with the indicated antibodies. (**F**,**G**) PMA-differentiated THP-1 cells were cotreated with the indicated concentrations of I3MT-3, or VX-765 as a positive control, and 200 μg/mL Alum for 4 h (**F**), or 3 μg/mL Poly dA:dT for 4 h (**G**). IL-1β release was analyzed by ELISA. Data shown are the mean ± S.D. Significant differences were determined by one-way ANOVA, followed by the Tukey–Kramer test; *** *p* < 0.001, ** *p* < 0.01, * *p* < 0.05, N.S. not significant. (**H**) PMA-differentiated THP-1 cells were pretreated with the indicated concentrations of I3MT-3 for 1 h and then treated with 1 μM Talabostat for 3 h. IL-1β release was analyzed by ELISA. Data shown are the mean ± S.D. Significant differences were determined by one-way ANOVA, followed by the Tukey–Kramer test; *** *p* < 0.001, ** *p* < 0.01. (**I**,**J**) BMDMs were treated with the indicated concentrations of I3MT-3 and 100 ng/mL LPS for 4 h, and then treated with 5 μM nigericin for 1.5 h (**I**), or 1 mM ATP for 1.5 h (**J**). IL-1β release was analyzed by ELISA. Data shown are the mean ± S.D. Significant differences were determined by one-way ANOVA, followed by the Tukey–Kramer test; *** *p* < 0.001. (**K**) The inhibitory effect of I3MT-3 on inflammatory cell death. PMA-differentiated THP-1 cells were cotreated with the indicated concentrations of I3MT-3 and 200 μg/mL Alum for 4 h. Cell cytotoxicity was measured by LDH release assay. Data shown are the mean ± S.D. Significant differences were determined by one-way ANOVA, followed by the Tukey–Kramer test; *** *p* < 0.001, ** *p* < 0.01. (**L**) Mice were treated with 1 µg LPS and 20 mg/kg I3MT-3 by intraperitoneal injection (IP) for 2 h, and then treated with 20 mg/kg gefitinib by IP. After 1 h, peritoneal lavage fluid was collected, and IL-1β levels were analyzed by ELISA. Data shown are the mean ± S.D. (*n* = 3). Significant differences were determined by one-way ANOVA, followed by the Tukey–Kramer test; ** *p* < 0.01, * *p* < 0.05.

In this study, we unexpectedly found that I3MT-3 inhibits mature IL-1β release induced by aluminum hydroxide (Alum) and polymyxin B (PMB), ligands that activate the NLRP3 inflammasome, in human monocytic THP-1 cells, in which phorbol myristate acetate (PMA) was primed to differentiate into macrophages (PMA-primed THP-1 cells) (Figure 1A–C) [20]. Moreover, the mature IL-1β release induced by Poly dA:dT or Talabostat, which activate the AIM2 or NLRP1 inflammasome, respectively, was clearly inhibited by I3MT-3 (Figure 1D,E). Meanwhile, results from an enzyme-linked immunosorbent assay (ELISA) confirmed that when Alum, Poly dA:dT, or Talabostat were used to treat PMA-primed THP-1 cells, I3MT-3 inhibited mature IL-1β release (Figure 1F–H). Similar results were observed when the NLRP3 inflammasome was activated by nigericin or ATP in mouse bone marrow-derived macrophages (BMDMs) primed by lipopolysaccharides (LPS), a toll-like receptor 4 (TLR4) ligand that transcriptionally upregulates pro-IL-1β expression (Figure 1I,J). We also observed that I3MT-3 inhibits Alum-induced cell death called pyroptosis (Figure 1K). As we have previously demonstrated that gefitinib, the tyrosine kinase inhibitor targeting the epidermal growth factor receptor, elevates the levels of mature IL-1β in peritoneal lavage through the NLRP3 inflammasome activation, we examined whether I3MT-3 inhibits mature IL-1β release induced by gefitinib in vivo [21]. As shown in Figure 1L, the levels of mature IL-1β in peritoneal lavage were significantly suppressed by I3MT-3. Taken together, these results suggest that I3MT-3 inhibits the activation of the NLRP3, AIM2, and NLRP1 inflammasomes.

### 2.2. I3MT-3 Inhibits Inflammasome Activation Independently of 3-MST Inhibition

**Figure 2:**
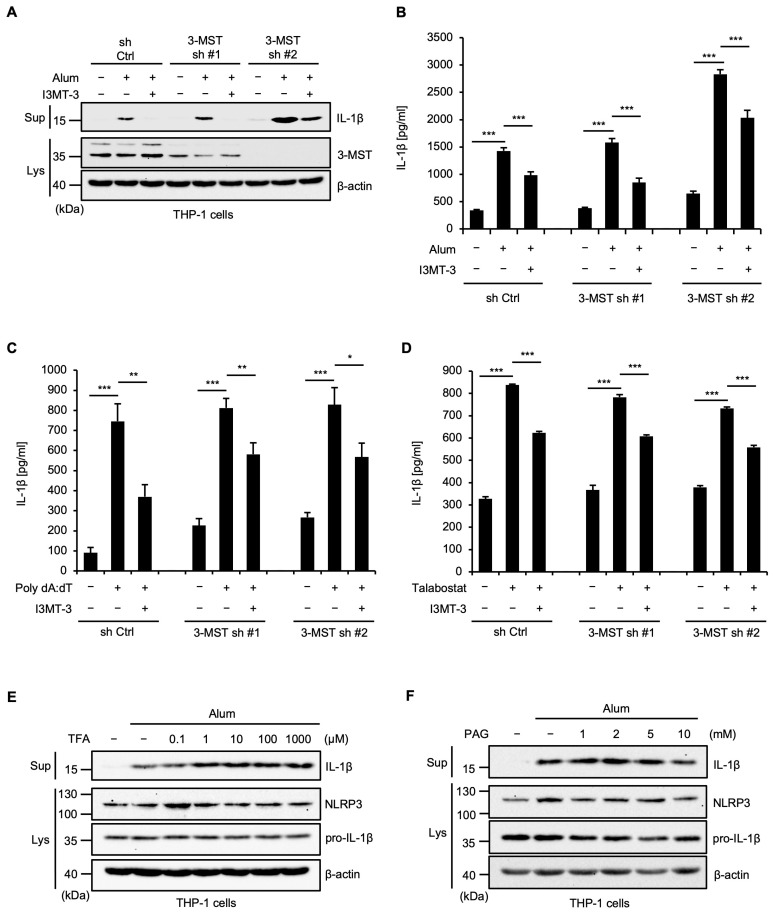
I3MT-3 inhibits inflammasome activation independently of 3-MST inhibition. (**A**) PMA-differentiated control or 3-MST KD THP-1 cells were cotreated with 50 μM I3MT-3 and 200 μg/mL Alum for 4 h. Cell-free supernatants (Sup) and cell lysates were subjected to immunoblotting with the indicated antibodies. (**B**,**C**) PMA-differentiated control or 3-MST KD THP-1 cells were cotreated with 50 μM I3MT-3 and 200 μg/mL Alum for 4 h (**B**), or 3 μg/mL Poly dA:dT for 4 h (**C**). IL-1β release was analyzed by ELISA. Data shown are the mean ± S.D. Significant differences were determined by one-way ANOVA, followed by the Tukey–Kramer test; *** *p* < 0.001, ** *p* < 0.01, * *p* < 0.05. (**D**) PMA-differentiated control or 3-MST KD THP-1 cells were pretreated with 50 μM I3MT-3 for 1 h and then treated with 1 μM Talabostat for 3 h. IL-1β release was analyzed by ELISA. Data shown are the mean ± S.D. Significant differences were determined by one-way ANOVA, followed by the Tukey–Kramer test; *** *p* < 0.001. (**E**,**F**) PMA-differentiated THP-1 cells were pretreated with the indicated concentrations of TFA (**E**) or PAG for 24 h (**F**), and then treated with 200 μg/mL Alum for 4 h. Cell-free supernatants (Sup) and cell lysates were subjected to immunoblotting with the indicated antibodies.

We next examined the involvement of 3-MST in the inhibition of inflammasome activation mediated by I3MT-3. To this end, we established 3-MST knockdown THP-1 cells as previously described [21]. As shown in Figure 2A,B, unexpectedly, the effect of I3MT-3 that inhibits mature IL-1β release induced by the NLRP3 inflammasome activation was observed in 3-MST knockdown THP-1 cells. Moreover, I3MT-3 inhibited the AIM2 and NLRP1 inflammasome activation in 3-MST knockdown THP-1 cells (Figure 2C,D). These observations therefore suggest that I3MT-3 inhibits inflammasome activation independently of 3-MST inhibition. On the other hand, we tested whether the inhibitors of other supersulfide-producing enzymes, such as CBS and CSE, inhibit inflammasome activation. As shown in Figure 2E,F, unlike I3MT-3, both the CBS inhibitor trifluoroalanine (TFA) and the CSE inhibitor DL-propargylglycine (PAG) failed to inhibit the mature IL-1β release induced by Alum. Therefore, among the inhibitors of supersulfide-producing enzymes, only I3MT-3 appears to possess the ability to inhibit the activation of the inflammasomes.

### 2.3. I3MT-3 Does Not Affect the Transcriptional Upregulation of Pro-IL-1β

**Figure 3:**
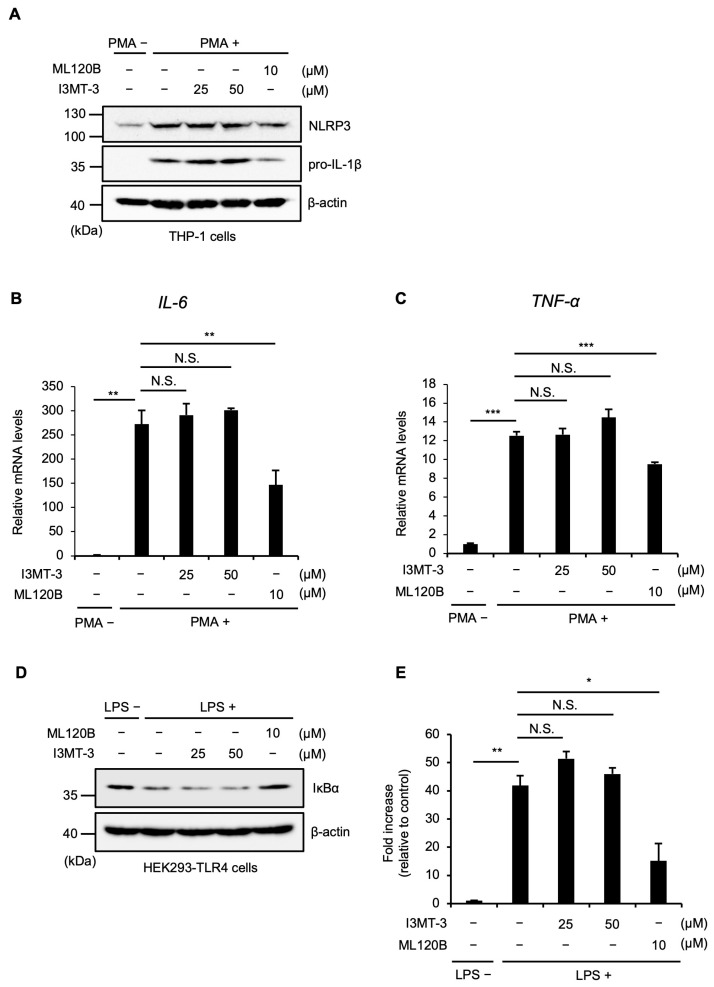
I3MT-3 does not affect the transcriptional upregulation of pro-IL-1β. (**A**) PMA-differentiated THP-1 cells were treated with the indicated concentrations of I3MT-3 or 10 μM ML120B for 6 h. Cell lysates were subjected to immunoblotting with the indicated antibodies. (**B**,**C**) PMA-differentiated THP-1 cells were treated with the indicated concentrations of I3MT-3 or 10 μM ML120B for 6 h. qRT-PCR was performed, with relative mRNA levels of IL-6 (**B**) or TNF-α (**C**). Data shown are the mean ± S.D. Significant differences were determined by one-way ANOVA, followed by the Tukey–Kramer test; *** *p* < 0.001, ** *p* < 0.01, N.S. not significant. (**D**) HEK293-TLR4 cells were pretreated with the indicated concentrations of I3MT-3 or 10 μM ML120B for 4 h and then treated with 100 ng/mL LPS for 4 h. Cell lysates were subjected to immunoblotting with the indicated antibodies. (**E**) HEK293-TLR4 cells were transfected with a plasmid and a Renilla luciferase plasmid for normalization. After 24 h, cells were pretreated with the indicated concentrations of I3MT-3 or 10 μM ML120B for 4 h and then treated with 100 ng/mL LPS for 4 h. Firefly and Renilla luciferase activities were quantified with a dual-luciferase assay kit. Data shown are the mean ± S.D. Significant differences were determined by one-way ANOVA, followed by the Tukey–Kramer test; ** *p* < 0.01, * *p* < 0.05, N.S. not significant.

Since the transcriptional upregulation of pro-IL-1β is critical for mature IL-1β release, we next examined whether I3MT-3 affects pro-IL-1β expression at the transcriptional level. It is well known that the activation of the nuclear factor-κB (NF-κB) signaling mediated by IκB kinase-β (IKKβ) leads to the upregulation of pro-IL-1β [23]. However, we found that I3MT-3 fails to inhibit the upregulation of pro-IL-1β induced by PMA, whereas N-(6-chloro-7-methoxy-9H-beta-carbolin-8-yl)-2-methylnicotinamide (ML120B), a selective and potent inhibitor of IKKβ, clearly achieved it (Figure 3A). In addition, I3MT-3 failed to inhibit the upregulation of IL-6 and tumor necrosis factor-α (TNF-α) (Figure 3B,C). Consistent with these results, I3MT-3 did not affect the degradation of IκBα induced by IKKβ activation (Figure 3D), and luciferase reporter assays of NF-κB revealed that I3MT-3 did not affect the reporter activity (Figure 3E). Therefore, these results suggest that I3MT-3 inhibits inflammasome activation through other mechanisms rather than the IKKβ-mediated NF-κB activation.

### 2.4. I3MT-3 Exerts an Inhibitory Effect on Inflammasome Activation by Targeting Caspase-1 but Not ASC

**Figure 4:**
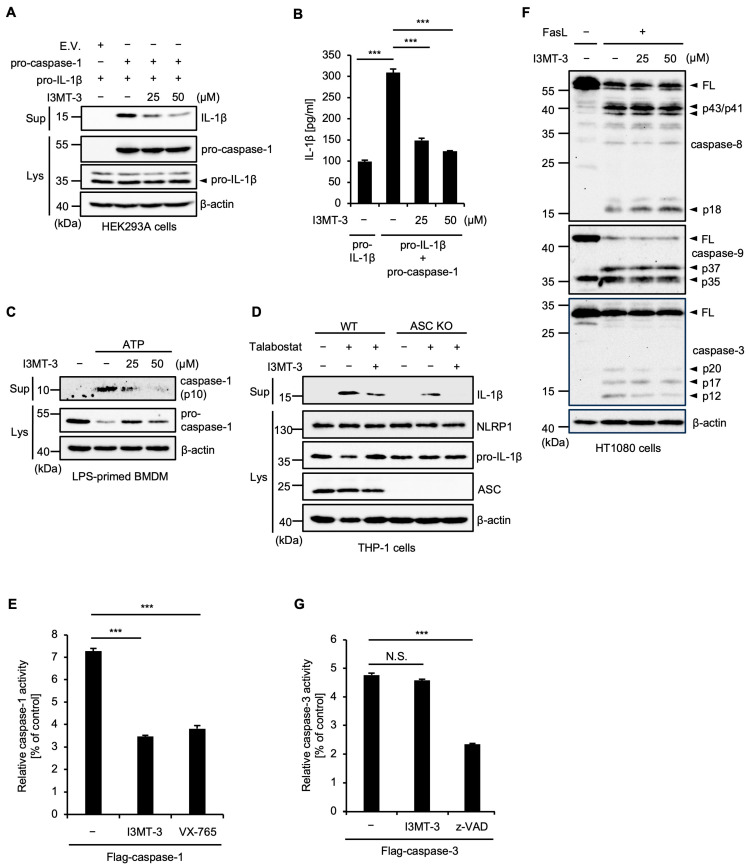
I3MT-3 exerts an inhibitory effect on inflammasome activation by targeting caspase-1 but not ASC. (**A**,**B**) HEK293A cells were transfected with plasmids of pro-caspase-1 and pro-IL-1β for 6 h, followed by overnight treatment with the above concentrations of I3MT-3. Cell-free supernatants (Sup) and cell lysates were subjected to immunoblotting with the indicated antibodies (**A**). IL-1β release was analyzed by ELISA (**B**). Data shown are the mean ± S.D. Significant differences were determined by one-way ANOVA, followed by the Tukey–Kramer test; *** *p* < 0.001. (**C**) BMDMs were treated with the indicated concentrations of I3MT-3 and 100 ng/mL LPS for 4 h, and then treated with 1 mM ATP for 1.5 h. Cell-free supernatants (Sup) and cell lysates were subjected to immunoblotting with the indicated antibodies. (**D**) PMA-differentiated ASC KO THP-1 cells were pretreated with 50 µM I3MT-3 for 1 h and then treated with 1 μM Talabostat for 3 h. Cell-free supernatants (Sup) and cell lysates were subjected to immunoblotting with the indicated antibodies. (**E**) HEK293A cells were transfected with plasmids of Flag-pro-caspase-1 for 24 h and then treated with 50 μM I3MT-3 or 50 μM VX-765 for 4 h. Purified Flag-caspase-1 was incubated for 20 min at RT in assay buffer. Then Ac-WEHD-pNA Colorimetric substrate 100 µM was added, and it was incubated at 37 °C for 2 h. Activity was measured using a microplate reader and the absorbance was read at 405 nm. Data shown are the mean ± S.D. Significant differences were determined by one-way ANOVA, followed by the Tukey–Kramer test; *** *p* < 0.001. (**F**) HT1080 cells were pretreated with the indicated concentrations of I3MT-3 for 24 h and then treated with 50 ng/mL FasL for 4 h. Cell lysates were subjected to immunoblotting with the indicated antibodies. (**G**) HEK293A cells were transfected with plasmids of Flag-pro-caspase-3 for 24 h and then treated with 50 μM I3MT-3 or 20 μM z-VAD-fmk for 4 h. Caspase-3 activity was measured by the Colorimetric caspase-3 assay. Data are shown as the ratio of caspase-3 activity versus the corresponding controls. Data shown are the mean ± S.D. Significant differences were determined by one-way ANOVA, followed by the Tukey–Kramer test; *** *p* < 0.001, N.S. not significant.

To further analyze the inhibitory mechanism of I3MT-3 for inflammasome activation, we focused on the activation of caspase-1, a common intracellular event downstream of inflammasome activation, because I3MT-3 shows an inhibitory effect regardless of the type of inflammasome. We first evaluated the effect of I3MT-3 on mature IL-1β release under conditions that induce the self-cleavage of caspase-1 independent of inflammasome formation, as previously demonstrated [24]. Interestingly, both immunoblot analysis and ELISA revealed that I3MT-3 inhibits caspase-1-dependent IL-1β release without inflammasome formation (Figure 4A,B). In addition, I3MT-3 clearly inhibited the caspase-1 processing (activation) induced by ATP in BMDMs, suggesting that I3MT-3 inhibits caspase-1 activation (Figure 4C). On the other hand, since NLRP1 possesses a caspase recruitment domain (CARD), NLRP1 can directly bind caspase-1 and then form the inflammasome that induces the cleavage and activation of caspase-1, even in the absence of ASC [25]. Indeed, we observed the mature IL-1β release induced by Talabostat in ASC knockout (KO) cells (Figure 4D). Moreover, we observed the inhibitory effect of I3MT-3 on the NLRP1 inflammasome activation in ASC KO cells (Figure 4D). Therefore, I3MT-3 appears to exert its inhibitory effect on inflammasome activation by targeting caspase-1 but not ASC. To examine whether I3MT-3 directly inhibits caspase-1 activation, we performed an in vitro caspase assay by using purified caspase-1 and a specific fluorescent substrate of caspase-1. As shown in Figure 4E, I3MT-3 clearly inhibited the caspase-1 activation in vitro, similar to the performance of the caspase-1 inhibitor VX-765 [22]. On the other hand, I3MT-3 failed to inhibit the caspase-8, -9, and -3 activation induced by Fas ligand (FasL) in human fibrosarcoma HT1080 cells (Figure 4F), and the caspase-3 activation in vitro (Figure 4G). Taken together, these results suggest that I3MT-3 inhibits caspase-1 but not other caspases through direct binding to caspase-1.

### 2.5. Molecular Docking Simulation of the Binding of I3MT-3 to Caspase-1

**Figure 5:**
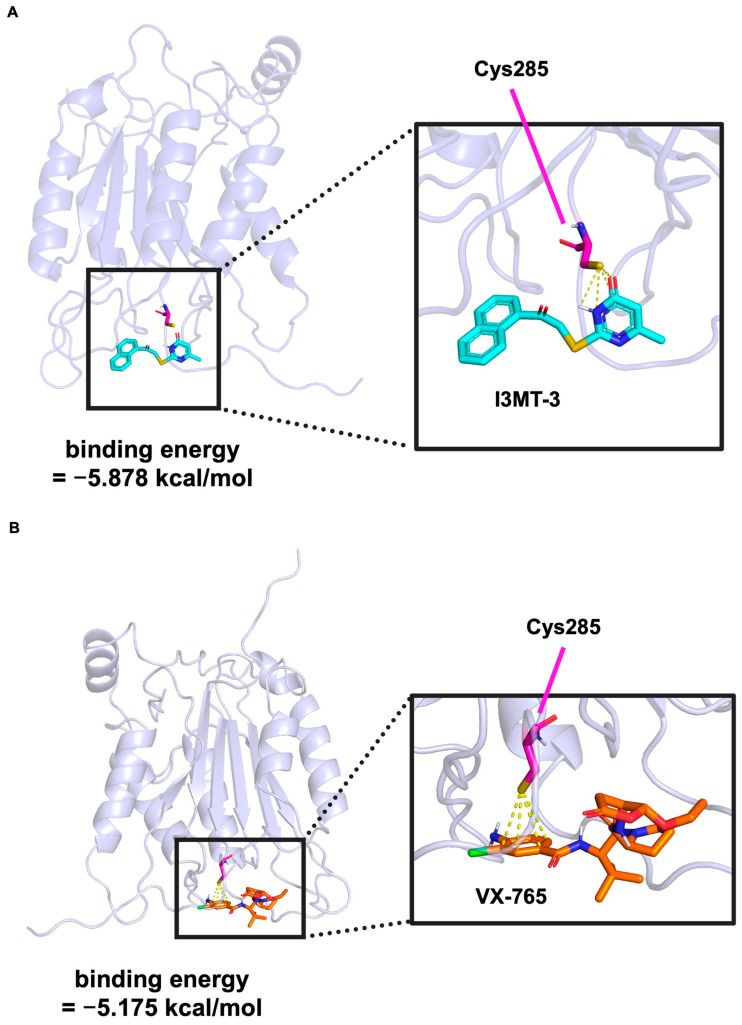
(**A**,**B**) Molecular docking to predict the binding of I3MT-3 to caspase-1 via AutoDock Vina, the results were visualized by PyMOL. The predicted binding conformation of caspase-1-I3MT-3 (**A**) and of caspase-1-VX-765 (**B**) are shown.

**Figure 6:**
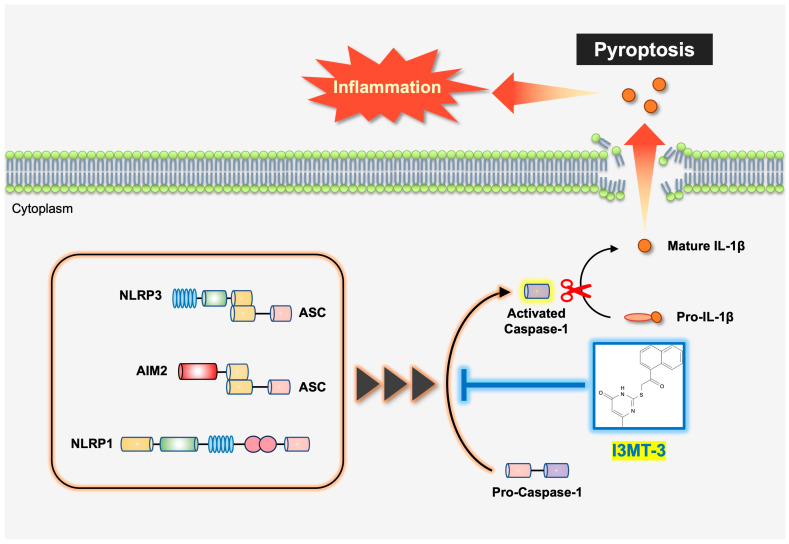
Schematic model to explain our study. I3MT-3 inhibits mature IL-1β release and pyroptosis associated with inflammasome activation. Mechanistically, I3MT-3 selectively inhibits the activity of caspase-1, an essential protein common to all inflammasomes such as NLRP3, AIM2, and NLRP1, and thereby suppresses a wide range of inflammatory stimuli.

It has been reported that electrostatic interaction between the pyrimidone ring of I3MT-3 and the sulfur atom of 3-MST is important when I3MT-3 inhibits 3-MST [19]. To investigate the inhibitory effect of I3MT-3 on caspase-1 activity in detail, we performed docking studies using AutoDock Vina based on the crystal structure of caspase-1 (PDB 6PZP) [26,27]. The active center of caspase-1 is composed of histidine at position 237 (H237) and cysteine at position 285 (C285), which are essential for its catalytic activity, and the thiol group of C285 is particularly important for its substrate cleavage [28,29]. As shown in Figure 5A, molecular docking simulations showed that I3MT-3 bound to C285 of caspase-1 (binding energy −5.878 kcal/mol). This result raises the possibility that the C=O and N-H groups in the pyrimidone ring of I3MT-3 may stabilize the bond with the thiol group in C285 of caspase-1 through electrostatic or polar interactions, since a stable bond can generally be formed if the binding energy is approximately less than −2.85 kcal/mol, as previously described [27]. Thus, the molecular docking simulations provided a low energy model in which I3MT-3 binds to the active center of caspase-1, and the pyrimidone ring in I3MT-3 stabilizes its binding. We performed a similar molecular docking simulation for the caspase-1 inhibitor VX-765, and then obtained a pose similar to PDB 6PZP (binding energy −5.175 kcal/mol), although it was slightly different from that obtained by the actual crystal structure analysis (https://www.wwpdb.org/pdb?id=pdb_00006pzp, accessed on 1 January 2025) (Figure 5B). From this binding energy value, the binding affinity of I3MT-3 for caspase-1 is relatively close to that of VX-765 for caspase-1. Therefore, it was assumed that I3MT-3 has the potential to function as a caspase-1 inhibitor. Taken together, these data suggest that I3MT-3 inhibits the activation of the NLRP3, AIM2, and NLRP1 inflammasomes by direct binding to caspase-1, and then mature IL-1β release (Figure 6).

## 3. Discussion

Anti-inflammatory drugs, such as the IL-1 receptor antagonists, are limited in that they cannot directly suppress inflammasome activation, and therefore are unable to suppress pyroptosis and the release of other cytokines activated by the inflammasomes [30]. On the other hand, specific inhibitors targeting the NLRP3 inflammasome (such as MCC950 and CY-09) have been developed, but none have been put to clinical use at present [31]. Furthermore, even if NLRP3 is specifically inhibited, it is difficult to completely suppress inflammation induced by other types of inflammasomes. In addition, there are increasing reports that various types of inflammasomes are associated with human diseases, so there is a need to develop drugs that suppress the activation of multiple inflammasomes [8,32,33]. In this study, we demonstrated that I3MT-3 suppresses the inflammation induced not only by NLRP3 but also by NLRP1 and AIM2. Given that caspase-1 is essential for the activation of multiple inflammasomes, caspase-1 inhibitors such as I3MT-3 have the potential to suppress a broad range of inflammatory responses.

Although this study demonstrated that I3MT-3 inhibits the activity of caspase-1, the specific structure and inhibitory mechanism of I3MT-3, which is important in inhibiting caspase-1 activity, remain unclear. In addition, since I3MT-3 was originally identified as the 3-MST inhibitor [19], it cannot be said to be selective for caspase-1. For this reason, it is important to identify the structural elements that contribute to the inhibition of caspase-1 activity, in order to make this study more valuable and increase potential of I3MT-3 as a therapeutic drug. Future studies that precisely analyze the interaction between I3MT-3 and caspase-1 at the molecular level may be able to identify the structural elements that serve as the basis for controlling caspase-1 activity. Based on our results, it is expected that more selective inhibitors for caspase-1 can be designed and developed.

## 4. Materials and Methods

### 4.1. Cell Lines

Human monocytic leukemia cell line THP-1 and human fibrosarcoma cell line HT1080 were obtained from the JCRB Cell Bank (Japanese Collection of Research Bioresources Cell Bank) [20]. Human embryonic kidney 293A (HEK293A) was from ATCC. THP-1 cells were cultured in RPMI 1640 containing 10% heat-inactivated FBS at 37 °C under a 5% CO_2_ atmosphere. For the experiments, THP-1 cells were differentiated for 3 h with 100 nM PMA before stimulation. HT1080 cells and HEK293A cells were cultured in DMEM containing 10% or 5% heat-inactivated FBS and 1% penicillin–streptomycin solution at 37 °C under a 5% CO_2_ atmosphere. Bone marrow-derived macrophages (BMDMs) were isolated from mouse femurs and were cultured in RPMI 1640 containing 10 ng/mL M-CSF, 10% heat-inactivated FBS, and 1% penicillin–streptomycin solution at 37 °C under a 5% CO_2_ atmosphere. BMDMs were primed with 100 ng/mL LPS.

### 4.2. Reagents and Plasmids

All reagents were obtained from commercial sources; dimethyl sulfoxide (DMSO), polymyxin B, ATP, gefitinib (Wako, Osaka, Japan), PMA, nigericin (Santa Cruz, Dallas, TX, USA), I3MT-3, VX-765 (MedChemExpress, Monmouth Junction, NJ, USA), Alum (Thermo Scientific, Waltham, MA, USA), M-CSF (Sino Biological, Beijing, China), Poly dA:dT, Ultrapure LPS (Invivogen, San Diego, CA, USA), Talabostat (Selleck, Houston, TX, USA), ML120B, TFA, PAG (Sigma-Aldrich, St. Louis, MO, USA), FasL (Enzo Life Sciences, Farmingdale, NY, USA), and z-VAD-fmk (Peptide Institute, Osaka, Japan). cDNAs encoding human pro-caspase-1, pro-IL-1β, and pro-caspase-3 were obtained by performing PCR, and were inserted into pcDNA3.2 with Flag tag plasmid as previously described [24]. Plasmid transfection was performed using Polyethylenimine “Max” (PEI-MAX) (Polysciences, Warrington, PA, USA), according to the manufacturer’s instructions.

### 4.3. Antibodies

Antibodies against the following proteins were used in this study; IL-1β (Cell Signaling Technology, Danvers, MA, USA, #83186/#12242), NLRP3 (AdipoGen Life Sciences, San Diego, CA, USA, AG-20B-0014), AIM2 (Santa Cruz, sc-515514), NLRP1 (Enzo Life Sciences, ALX-210-018), IκBα (#9242), 3-MST (sc-376168), caspase-1 (sc-622/sc-514), ASC (sc-514414), caspase-8 (ALX-804-242), caspase-9 (#9508S), caspase-3 (sc-271028), and β-actin (sc-47778). Mouse IgG, rat IgG, and rabbit IgG (Cell Signaling Technology) were also used in this study.

### 4.4. Generation of Knockout Cell Lines

ASC knockout cells were established using the CRISPR/Cas9 system as previously described [19]. Guide RNAs (gRNAs) were designed to target a region in the exon 1 of the ASC gene (5′-CAGCACGTTAGCGGTGAGCT-3′) using CRISPRdirect. gRNA-encoding oligonucleotide was cloned into lentiCRISPRv2 plasmid (Addgene, Watertown, MA, USA), and knockout cells were established as previously described [21]. To determine the mutations of each gene in cloned cells, the genomic sequence around the target region was analyzed by PCR-direct sequencing using extracted DNA from each clone as a template and the following primers: 5′-TTGGACCTCACCGACAAG-3′ and 5′-GCAGCTTTGTTTAGGGGTAGG-3′ for ASC.

### 4.5. Generation of Knockdown Cell Lines

The 3-MST or control shRNA (pLKO.1) lentivirus vector (MISSON shRNA; Sigma-Aldrich) was transfected with the VSV-G envelope and psPAX2 packaging plasmids (Addgene) into HEK293A cells using PEI-MAX as previously described [19]. After 48 h, lentivirus-containing supernatants were harvested and centrifuged at 1000 rpm for 5 min to discard the debris. THP-1 cells were infected with the virus for 24 h and then selected by 1 μg/mL puromycin for 72 h. Knockdown efficacy was analyzed by immunoblotting.

### 4.6. Stable Cell Lines

Stable cell lines that express human TLR4 were established by retroviral transduction as follows [34]. Phoenix-AMPHO (the packaging cell line) was transfected with pMXs-IH inserted with cDNAs encoding human TLR4. After 48 h, the growth medium that contained retrovirus was collected. HEK293 cells were incubated with the (virus-containing) medium with 10 µg/mL polybrene for 48 h, and uninfected cells were eliminated through hygromycin selection.

### 4.7. Immunoblot

Proteins from cell culture supernatants were extracted by methanol/chloroform precipitation. In brief, cell-free supernatant was mixed with methanol/chloroform at a 5:5:1 (cell culture supernatant/methanol/chloroform) ratio. The mixture was vortexed and centrifuged for 10 min at 15,000 rpm. The clear upper phase was discarded, and 1000 μL of methanol was added to the interphase. The mixture was centrifuged for 10 min at 15,000 rpm, and the liquid phase was removed. The protein pellet was dried and resuspended with 8 M urea. The cells were lysed in ice-cold lysis buffer containing 20 mM Tris-HCl (pH 7.4), 150 mM NaCl, 10 mM EDTA, 1% Triton X-100, 1% sodium deoxycholate, and 1% protease and phosphatase inhibitor mixtures (Nacalai Tesque, Kyoto, Japan). The lysates were centrifuged at 15,000 rpm for 15 min. Samples were resolved by SDS-PAGE and analyzed as previously described [24].

### 4.8. Enzyme-Linked Immunosorbent Assay (ELISA)

After the indicated stimulation or treatment, the plates seeded with cells were centrifuged at 1500 rpm for 3 min. After centrifugation, the supernatants were collected and used as a sample for ELISA. The concentrations of IL-1β in supernatants of cell culture were measured by specific ELISA kits (Invitrogen, Waltham, MA, USA) according to the manufacturer’s instructions.

### 4.9. Cell Death Assay

Cell death was measured using a Lactate Dehydrogenase (LDH)-Cytotoxic Test Kit (Wako) according to the manufacturer’s protocol. The activity level of the LDH released into the culture media was quantified as a percentage of the total activity level of LDH as previously described [21]

### 4.10. Quantitative Real-Time PCR

Total RNA was extracted from culture cells and kidneys of mice using Sepasol-RNA I Super G (Nacalai Tesque) and reverse transcribed using a high-capacity cDNA reverse transcription kit (Applied Biosystems, Waltham, MA, USA) according to the manufacturer’s instructions. Template cDNA was amplified by quantitative real-time PCR as previously described [35]. Gene expression levels were normalized with that of GAPDH. The primers (human) used for quantitative real-time PCR were as follows: 5′-TACCCCCAGGAGAAGATTCC-3′ and 5′-TTTTCTGCCAGTGCCTCTTT-3′ for IL-6; 5′-CGAGTGACAAGCCTGTAGCC-3′ and 5′-TTGAAGAGGACCTGGGAGTAGATG-3′ for TNF-α; 5′-AACAGCCTCAAGATCATCAGC-3′ and 5′-GGATGATGTTCTGGAGAGCC-3′ for GAPDH.

### 4.11. Luciferase Assay

The reporter assays were performed essentially as previously described [35]. The cells were transfected with NF-κB-luc using PEI-MAX according to the manufacturer’s instructions. After 24 h, the cells were treated with I3MT-3 or ML120B, and then their luciferase activities were assayed using a dual-luciferase reporter assay system (Promega, Madison, WI, USA) according to the manufacturer’s instructions.

### 4.12. Colorimetric Caspase-3 Assay

HEK293A cells transfected with Flag-pro-caspase-3 were treated with I3MT-3 or z-VAD-fmk before collection. The cells were lysed in 100 µL of the cell lysis buffer included in the Caspase-3 Colorimetric Assay Kit (Biovision, Milpitas, CA, USA), and then the lysates were centrifuged at 15,000 rpm for 15 min. A total of 90 µL of the cell extracts was mixed with caspase reaction buffer (10 mM Tris-HCl pH 7.4, 150 mM NaCl, 0.1% CHAPS, 2 mM MgCl_2_, 5 mM EGTA, and 1 mM DTT) 10 µL supplemented with the caspase-3-specific substrate DEVD-pNA (Biovision) at a final concentration of 100 µM and incubated at 37 °C for 1 h. Its activity was measured using a microplate reader and the absorbance was read at 405 nm.

### 4.13. In Vitro Caspase-1 Assay

HEK293A cells were transfected with Flag-pro-caspase-1 and treated with I3MT-3 for 4 h before collection. The cells were lysed in ice-cold lysis buffer containing 20 mM Tris-HCl (pH 7.4), 150 mM NaCl, 10 mM EDTA, 1% Triton X-100, and 1% sodium deoxycholate, and then the cell lysates were centrifuged at 4 °C at 15,000 rpm for 15 min. Their supernatants were immunoprecipitated with anti-Flag antibodies (anti-Flag affinity M2 gel; Sigma-Aldrich) and eluted with 250 μg/mL 3 × Flag-peptide (Sigma-Aldrich). Purified Flag-caspase-1 was incubated for 20 min at RT in assay buffer (20 mM HEPES pH 7.5, 10 mM KCl, 1.5 mM MgCl_2_, 1 mM EDTA, 1 mM EGTA, and 1 mM DTT). Then Ac-WEHD-pNA Colorimetric substrate (Abcam, Cambridge, UK) 100 µM was added, and it was incubated at 37 °C for 2 h. The activity was measured using a microplate reader and the absorbance was read at 405 nm.

### 4.14. Ligand and Protein Preparation

The structures of I3MT-3 and VX-765 as ligands were obtained from PubChem (https://pubchem.ncbi.nlm.nih.gov, accessed on 1 January 2025) as SDF format. Next, using Avogadro (version 1.2.0), the optimized structures were converted to Protein Data Bank (PDB) format. Gasteiger charges were added using AutoDockTools (version 1.5.7) [36,37]. In addition, when setting the number of rotatable bonds of the ligand, all rotatable bonds were set to be rotatable. The structures were then converted to PDBQT (Protein Data Bank, Partial Charge (Q), and Atom Type (T)) format using AutoDock Vina [26,27,38]. The structure of caspase-1 in complex with VX-765 (PDB ID: 6PZP) was obtained from PDB (https://www.rcsb.org, accessed on 1 January 2025) in PDB format as a receptor protein that docks with ligands. Water molecules and initial ligands were removed from the receptor protein structure using PyMOL (version 3.0.0). Finally, the prepared protein structure was saved in PDBQT format for use in docking simulations.

### 4.15. Docking Simulations

Molecular docking simulations were performed using AutoDock Vina (version 1.2.5) to predict the binding affinity and pose of I3MT-3 or VX-765 to caspase-1. The grid box for specifying the docking site between caspase-1 and the ligands was set so as to surround the entire receptor molecule. The docking configuration file included the center coordinates and size of the grid box, with the spacing between the grid points set to 0.375 Å, the exhaustiveness set to 32, the number of modes set to 100, and the energy range set to 3. Molecular docking was performed using AutoDock Vina (version 1.2.5) to predict the binding affinity and binding pose of the ligand and receptor protein.

### 4.16. Visualization and Analysis

The docking results were visualized and analyzed using PyMOL (version 3.0.0). Interactions between the ligand and the protein were examined to identify key binding residues, and the binding energy values were compared to assess the stability of the ligand–protein complex.

### 4.17. Animal Experiments

C57BL/6J mice were obtained from CLEA Japan (Tokyo, Japan). The mice were maintained according to the Guidelines for Animal Experimentation of Tohoku University, and all the procedures were approved by the Institutional Animal Care and Use Committee at Tohoku University (approval number: 2019PhA-026). For IL-1β evaluation, 8-week-old C57BL/6 (male) mice were treated with 1 µg of LPS and 20 mg/kg I3MT-3 in PBS/dimethyl sulfoxide (DMSO) by IP. After 2 h, the mice were treated with 20 mg/kg gefitinib in PBS/DMSO by IP. After 1 h, the peritoneal cavities were lavaged with ice-cold PBS, and then heparin and protease inhibitor cocktail were added to the peritoneal lavage fluid. The collected peritoneal lavage fluid was centrifuged for 3 min at 5000 rpm to separate fluid from cells. IL-1β levels in the supernatants were analyzed by ELISA.

### 4.18. Statistical Analysis

The value was described as the mean ± standard deviation (SD) using Prism 9 Version 9.5.1 software (GraphPad, La Jolla, CA, USA). Multiple-group comparisons were conducted using one-way ANOVA analysis of variance followed by the Tukey–Kramer test using Prism software (GraphPad). Data were considered significant when *** *p* < 0.001, ** *p* < 0.01 and * *p* < 0.05. All experiments were repeated at least three independent times.

## Data Availability

The data presented in this study are available in the article.
